# Hippocampal population dynamics underlying memory trace activation in a tactile classification task

**DOI:** 10.1186/1471-2202-12-S1-P70

**Published:** 2011-07-18

**Authors:** Athena Akrami, Pavel Itskov, Mathew E Diamond

**Affiliations:** 1 Cognitive Neuroscience Sector, SISSA, Trieste Italy

## 

Hippocampal neuronal ensemble activity appears to play an important role in establishment of spatial and non-spatial episodic memories [1] and [2]. In particular, CA1 region of the hippocampus is well known to be a crucial site for processing associative memories which typically contain information about what, where, and when major behavioral events occurred [3]. In simple navigation tasks, it was shown that different spatial information can unfold in time either by complete reorganization of hippocampal place code in which both place and rate of firing take statistically independent values (global remapping, [4] and[5]) or instead, through minor modulation of firing rates in a same neural assembly (rate remapping, [4] and [5]). But to date, there is no evidence how CA1 ensembles remap to code different phases of a sensory, in particular tactile, memory task, at population level. Previously, it was shown how “single” cells in CA1 adapt an independent coding to represent tactile and reward location information [6]. This finding indicates that hippocampal neurons did not encode textured stimuli as physical objects along specific dimensions (e.g. coarseness), but as meaningful events in conjunction with the location in which they appeared. Moreover, the coding properties of neurons did not “follow” them across contexts. Here, we further analyzed population dynamics underlying this independence coding and explore both quantitative and qualitative differences between information content of single unit activities versus population vector. On each trial the rat touched a textured plate with its whiskers, and then turned towards the Left or Right water spout.

Firstly, we measured the degree of rate-remapping, due to task-induced changes in population activity. The kolmogorov-smirnov distance between the empirical firing rate distribution of each single cell, collapsed in time and trial, and different null hypotheses, based on different assumptions on global remapping was computed. We also computed the correlation between pairs of population vectors taken from different times of each trial. These measures are independent of the relative magnitude of firing. Comparing the distributions of correlation values for entire set of population vectors between two trials, or two behaviorally different phases of each trial, provides an estimate of how much the ensemble code changes. When the rats experienced different phases of the task, however, the hippocampus encoded these changes robustly by changing the values of the components of the population vectors, without much, if any, change in the ensemble of recruited cells. Secondly, to see how presence of noise correlations may enhance coding property of the population, the performance of a non-linear classifier trained on few informative “single” cells was contrasted with a classifier trained on a) the complete set of cells and b) the principle components of the cell assembly. Thirdly, we applied trajectory-based activity classification of neural responses using Hidden Markov models, to segment the complete recording into a sequence of a few statistically discriminated hidden states, each correlated with different behavioral condition.
